# Laboratory markers included in the Corona Score can identify false negative results on COVID-19 RT-PCR in the emergency room

**DOI:** 10.11613/BM.2020.030402

**Published:** 2020-08-05

**Authors:** Roberto Assandri, Ciro Canetta, Giovanni Viganò, Elisabetta Buscarini, Alessandro Scartabellati, Alessandro Montanelli

**Affiliations:** 1 Clinical Investigation Laboratory, ASST-Crema, Maggiore Hospital, Crema, Italy; 2 Internal Medicine Unit, ASST-Crema, Maggiore Hospital, Crema, Italy; 3 Emergency Medicine Unit,ASST-Crema, Maggiore Hospital, Crema, Italy; 4 Gastroenterology Department, ASST-Crema, Maggiore Hospital, Crema, Italy; 5 Pneumology 2 Department, ASST-Crema Maggiore Hospital, Crema, Italy; 6 Clinical Investigation Laboratory, ASST-Bergamo Est, Bolognini Hospital Seriate, Bergamo, Italy

**Keywords:** COVID-19, Emergency room, RT-PCR, laboratory markers, Corona score

## Abstract

After December 2019 outbreak in China, the novel Coronavirus infection (COVID-19) has very quickly overflowed worldwide. Infection causes a clinical syndrome encompassing a wide range of clinical features, from asymptomatic or oligosymptomatic course to acute respiratory distress and death. In a very recent work we preliminarily observed that several laboratory tests have been shown as characteristically altered in COVID-19. We aimed to use the Corona score, a validated point-based algorithm to predict the likelihood of COVID-19 infection in patients presenting at the Emergency rooms. This approach combines chest images-relative score and several laboratory parameters to classify emergency room patients. Corona score accuracy was satisfactory, increasing the detection of positive patients’ rate.

After December 2019 outbreak in China, the novel Coronavirus infection (COVID-19) has very quickly overflowed worldwide. Infection causes a clinical syndrome encompassing a wide range of clinical features, from asymptomatic or oligosymptomatic course to acute respiratory distress and death (*1*, *2*). As of April, 2020 a total of 104,291 laboratory-confirmed cases have been documented in Italy, and Lombardy, the Northern Italian Region, recorded over 60,000 COVID-19 cases. Maggiore Hospital of Crema began one of the Italian battlefronts. Frequency of disease and fatality rate are calculated on the number of patients positive to oral, nasal, or nasopharyngeal swab. However, the European Community, Schengen area and also Italian Regions had released different policies to define the use of swab and real-time reverse transcription polymerase chain reaction (RT-PCR) as diagnostic tools. Population-scale testing for COVID-19 is one of the best ways to limit mortality rates. Large-scale testing finds and isolates infections quickly, limiting the virus’ spread and protecting vulnerable populations. Millions of COVID-19 test kits will need to be processed. Organizations around the world are trying to improve their capacity as quickly as possible, but the challenge is too hard. Local, National and International Media continuously attack Regional and National Institutions, complaining about the inability of the Lombardy Region, the most solid and advanced Italian Region in terms of public health, not to have the possibility to processing a capillary population based swab screening test. However, diagnostic test, mostly involving nasopharyngeal swab, can be inaccurate in two ways. A false positive result erroneously considers a person infected and consequently includes an unnecessary quarantine. False negative results can weight much more because of real affected person will be not isolate and can infect other ones.

In a very recent work we observed that several laboratory tests have been shown as characteristically altered in COVID-19 cases (*3*). Thus, we have proposed a number of laboratory tests as rapid and sensitive alternatives in identifying likely cases, during Emergency room activity (*4*). In our paper baseline biochemical parameters have been clearly linked to clinical features (*3*). Our haematological tests at admission showed low white blood cell count, low neutrophil count in over 80% of cases, and lymphocyte count below 1 x10^9^/L in over 55%. Also C-reactive protein (CRP) serum concentrations were higher in most patients, particularly among those with worst clinical presentation and outcome (*3*).

In the light of these observations we decided to choose an “*alternative*” viewpoint that combined microbiological and biochemical parameters to identify likely COVID-19 patients in our emergency room. We decided to use the Corona score, a validated point-based algorithm, to predict the likelihood of COVID-19 infection in patients presenting at the Emergency rooms (scale 0-14) (*5*). This approach uses chest images-relative score (1 to 4) and several laboratory parameters to classify emergency room patients (*5*).

In a new cohort of 240 Emergency room patients (from May 16^th^ to May 18^th^) we detected COVID-19 ribonucleic acid (RNA) and applied the Corona score. We used a cut-off value of 5 (sensitivity 94%, specificity 72%) with best accuracy, as previously described (*5*). Viral RNA was detected with the Simplexa COVID-19 Direct kit directly from swab specimens, by two genes amplification, *ORF1ab* and *S*.

Two different patient groups were detected, using RT-PCR as diagnostic criteria: RNA-negative (232 patients, 97%) with a median Corona Score 4 (0-10) and RNA-positive patients (8 patients, 3%) with a median Corona Score as 12 (5-10). RNA-negative Group showed six patients with high Corona score (2.6%, from 8 to 10). All patients were considered positive at computer tomography scan (CT-scan), based on multiple patchy ground glass shadows accompanied by septal thickening images. Nasopharyngeal swabs of these patients were re-tested with Xpert Xpress COVID-19 (*N* and *E* genes). All patients resulted positive, with amplification of both genes. So we considered positive fourteen patients (5.8% *versus* 3%). Corona score accuracy was satisfactory, increasing the detection of positive patients’ rate. Our study, according to previously published data, has identified a new and rapid tool to be implemented in the Emergency room.

As suggested by Kurstjens and co-workers, also confirmed in our study, the sensitivity of the Corona-score appears to exceed the sensitivity of the initial COVID-19 RT-PCR, which moreover demonstrated a specificity of 45% in our previous work (*3*, *5*).

However, several issues must be addressed. First, the appropriate procedure to obtain a nasopharyngeal swab specimen is essential, in order to minimize false negative results. Expertise and training of the person are necessary to obtain a right, representative collection. The quite low attention to swab procedure in emergency room is more than justified, especially during a period in which Emergency Department is literally “*under attack”.* However more cautions are needed. For these reasons the score can be used as interesting instrument, optimizing the predictive outcome of RT-PCR tests, clinical decisions and consequently patient’ isolation. Second, as suggested by Kurstjens, this algorithm is recommended to be used only in patients with respiratory symptoms (*5*). But we recently observed that gastrointestinal symptoms (GS) are present in 3-10% of hospitalized patients (*6*). Also we observed that GS were neither associated with fever or cough (*6*). This evidence can affect the sensibility of score, limiting the use to a non-comprehensive patients cohort.

We propose the use of the Corona score to assess the severity of COVID-19 in the Emergency room, and furthermore we propose a possible use of a modified Corona score in which the scale-associated to CT images could be eliminated; in this way we will be able to apply the score in all Emergency room patients (in patients with GS as well).
